# The Merendino procedure following preoperative imatinib mesylate for locally advanced gastrointestinal stromal tumor of the esophagogastric junction

**DOI:** 10.1186/1477-7819-6-37

**Published:** 2008-04-04

**Authors:** Wilko I Staiger, Ulrich Ronellenfitsch, Georg Kaehler, Hans Ulrich Schildhaus, Antonia Dimitrakopoulou-Strauss, Matthias HM Schwarzbach, Peter Hohenberger

**Affiliations:** 1 Div. Surgical Oncology and Thoracic Surgery, Department of Surgery, University Hospital Mannheim, Medical Faculty Mannheim, University of Heidelberg, Germany; 2 Department of Pathology, University of Bonn Medical School, Germany; 3 Medical PET Group – Biological Imaging, Clinical Cooperation Unit Nuclear Medicine, German Cancer Research Center, Heidelberg, Germany

## Abstract

**Background:**

Gastrointestinal stromal tumors (GIST) of the esophagogastric junction might pose a major problem to surgical resection. If locally advanced, extended or multivisceral resection with relevant procedural-specific morbidity and mortality is often necessary.

**Case presentation:**

We report a case of a patient with a locally advanced GIST of the esophagogastric junction who was treated by transhiatal resection of the lower esophagus and gastric cardia with reconstruction by interposition of segment of the jejunum (Merendino procedure). Prior to resection, downsizing of the tumor had successfully been achieved by treatment with imatinib mesylate for six months. Histological proof of GIST by immunohistochemical expression of c-KIT and/or PDGF alpha Receptor is crucial to allow embarking on this treatment strategy.

**Conclusion:**

A multimodal approach for an advanced GIST of the esophagogastric junction with preoperative administration of imatinib mesylate could avoid extended resection. The Merendino procedure might be considered as the reconstruction method of choice after resection of GIST at this location.

## Background

Gastrointestinal stromal tumors (GISTs), although relatively rare, are the most common mesenchymal tumors of the gastrointestinal (GI) tract. Recently, GISTs were defined by the characteristic expression of the c-Kit protooncogene (CD117) and specific histological and immunohistochemical criteria [1]. It has been postulated, that the so called interstitial cells of Cajal (ICC) are related to GIST tumors. ICCs are part of the autonomic nervous system regulating the peristalsis of the GI tract. Others hypothesize that GIST originate from primitive (stem) cells in the GI tract, which then can develop into an ICC [2].

The population-based annual incidence of GISTs is estimated with 14.5 per million for Sweden, but the figure may also contain GISTs detected incidentally and at autopsy [3]. In the SEER (Surveillance, Epidemiology and End Results) data from 1992 to 2000 the age-adjusted yearly incidence rate was 6.8 per million [4]. The median age at diagnosis has been reported to be 55 to 65 years [5]. The incidence of GIST is not known for all populations, most data refer to Caucasian industrialized populations.

In the past, surgery was the only effective treatment for localized GIST. Radiotherapy has been applied without success and the response rates of standard chemotherapy regimes in series published before 2000 have been very poor and could not prevent early relapse and tumor related death in metastasized patients. The introduction of the molecular-targeted therapeutic agent imatinib mesylate (STI571, Glivec^®^, Novartis) in 2001 significantly improved the outcome especially in patients with advanced and metastasized disease [6,7]. Imatinib mesylate is a selective small molecule receptor inhibitor of tyrosine kinases including c-Kit which was initially approved for the treatment of chronic myelogenous leukaemia harbouring c-Kit and BRC-ABL mutations. Following the demonstration of an objective response in more than 50% of the treated patients with GISTs, imatinib mesylate became rapidly the therapy of choice for unresectable or metastatic GIST [8]. The therapy is well tolerated with mild side effects diminishing with continuous treatment.

Although GIST can arise everywhere in the gastrointestinal tract, they most often occur in the stomach (50% to 60%). About 20% to 30% of GISTs develop in the small intestine. GIST of the gastroesophageal junction (GEJ) or distal esophagus are rare with less than 5% and have been described only in small series or case reports [9,10]. Small GISTs of the esophagus often are handled by gastroenterologists with an endoscopic surveillance strategy or sometimes endoscopic resection. Due to a main extraluminal growth GIST can reach a size of up to 15 cm prior to diagnosis. Then however, the tumors are often treated like carcinoma of the GEJ with extensive surgery and lymphadenectomy such as abdomino-thoracic or transhiatal esophageal resection. The preferred method of reconstruction is esophagogastric anastomosis with considerable subsequent morbidity [11,12]. As GISTs usually do not metastasize to lymph nodes, a less radical approach could be considered. However, large tumor size and peritumorous neoangiogenesis often make a limited but complete resection difficult. In this setting, cytoreduction and regression of the peritumorous neoangiogenesis through imatinib mesylate therapy with neoadjuvant intent may decrease the risk of tumor rupture and bleeding and increase the likelihood of potential curative resection.

We report the case of a patient with a locally advanced GIST of the GEJ who was first treated by imatinib mesylate followed by tumor removal with limited resection and interposition of a segment of the jejunum (Merendino procedure).

## Case Presentation

### Patient

**Figure 1:**
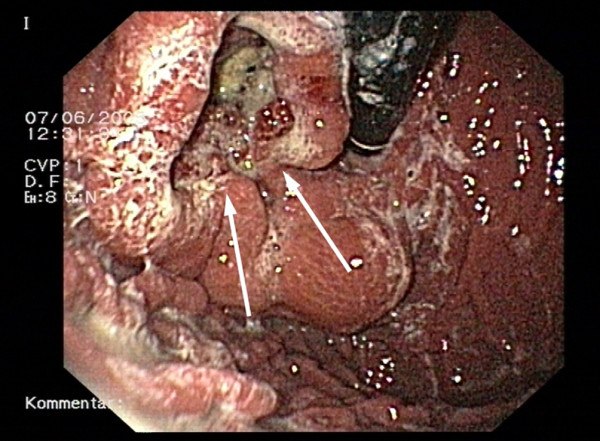
**Esophago-gastroscopy**. Preoperative esophago-gastroscopy, showing an ulcerous lesion of the esophagogastric junction.

**Figure 2:**
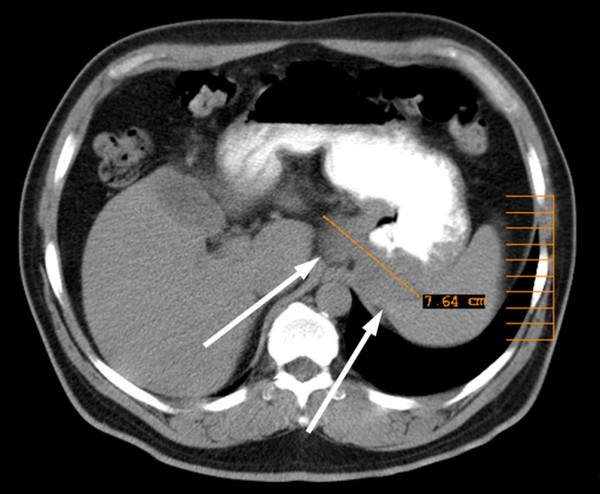
**Initial CT-Scan**. Initial CT scan showing the advanced tumor of the esophagogastric junction before starting neoadjuvant therapy.

A 51-year-old male was referred to our hospital with dysphagia and recurrent upper abdominal discomfort. Apart from arterial hypertension, no significant medical history was reported. Endoscopy detected an ulcerous lesion dorsal at the GEJ (figure 1), however, biopsies did not prove malignant disease. Deep biopsies lead to the histopathological diagnosis of a GIST in the GEJ. High-resolution multislice computerized tomography (CT) showed a solid tumor measuring 7.6 cm extending from the distal esophagus to the gastric cardia and fundus with extension into of the left diaphragmatic muscular column and the splenic hilus (figure 2). Surgery with curative intent at this stage would have required a multivisceral resection by an abdomino-thoracic approach, including resection of the left diaphragmatic muscle as well as splenectomy. After thorough discussion of the treatment options, the patient consented to try to downstage the tumor first by neoadjuvant treatment with imatinib mesylate followed by surgery after three to six months.

### Staging and neoadjuvant treatment

**Figure 3:**
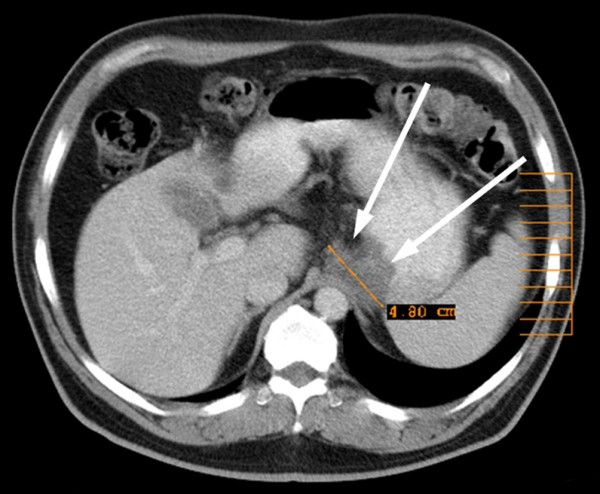
**Follow up CT-Scan**. Follow up CT scan after 6 months of treatment with imatinib mesylate, showing considerable regression of tumor.

Liver metastases were excluded by ultrasound and abdominal CT as were lung metastases by conventional chest x-ray and CT of the thorax. Before starting with drug treatment the patient underwent functional staging with ^18^F-FDG-PET demonstrating an increased tumor metabolism without signs of distant tumor spread. Imatinib mesylate at 400 mg per day was given orally. The patient suffered from mild diarrhoea and nausea during the treatment. The side effects were controlled by loperamide and metoclopramide. Follow-up ^18^FDG-PET examination two months after the beginning of the treatment showed a steep decline of ^18^FDG-uptake at the area of the tumor which by visual analysis of the PET could no longer be detected. This result documented response to treatment with imatinib mesylate which was continued for another four months. Follow-up CT at six months revealed a regression of the tumor diameter from 7.6 cm to 4.8 cm. The tumor margin showed a wash-out phenomenon with loss of contrast enhancement and no clear delineation (figure 3). Resection of the residual tumor was felt to be possible now with preservation of the distal stomach and the spleen.

### Operation

**Figure 4:**
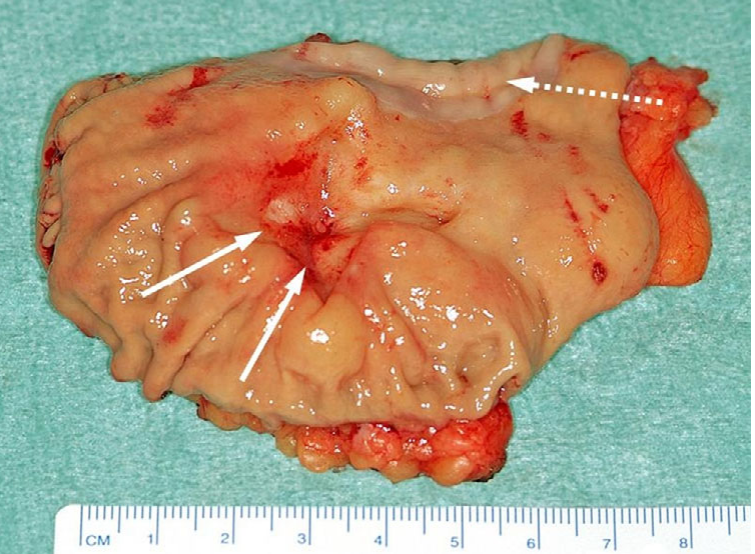
**Postoperative specimen**. Postoperative specimen showing the residual ulcerous lesion and esophageal mucosa in the upper part (interrupted arrow).

Intraoperatively the tumor was found to be located dorsal of the GEJ. The diaphragm was incised and after mobilisation of the greater and lesser curvature and opening of the lesser sac, the tumor could be mobilized easily from the pancreas as well as from the splenic hilus. Through mobilisation of the distal esophagus the tumor was resected en-block by linear stapler technique together with the gastric fundus and cardia using the retroperitoneal fat and parts of the left column of the diaphragm for covering the residual mass and as resection margin. The postoperative specimen showed residues of the ulcerous lesion (figure 4). For reconstruction of the food passage an isoperistaltic jejunal segment was inserted.

### Histopathological findings

**Figure 5:**
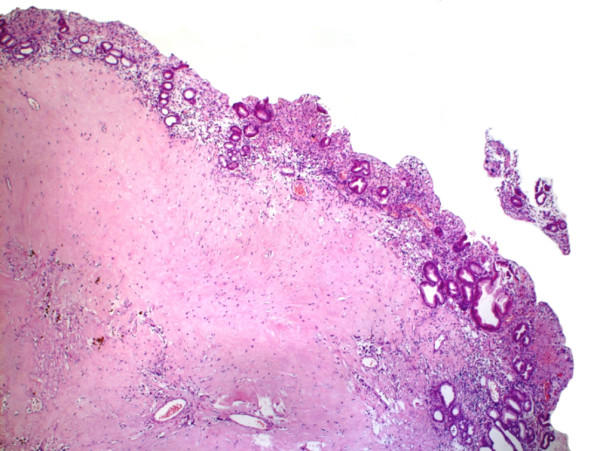
**Histological examination**. Postoperative histology with regressive changes under normal gastric mucosa.

Histopathological examination of the resection specimen confirmed a GIST with extensive regressive changes. The tumor originated from the submucosal layers and extended to the subserosa with a remaining diameter of 2.5 cm (figure 5). Tumor cells were still positive for c-Kit, but the proliferation rate measured with Ki-67 expression was less than 10%. Oral and aboral resection margins were free of tumor cells as were eight perigastric lymph nodes. Molecular pathology of exon mutation analysis could not find a mutation in exons 9 and 11 of c-Kit nor in exon 18 of PDGF receptor alpha. Thus the case was classified as 'wildtype'.

### Postoperative course

**Figure 6:**
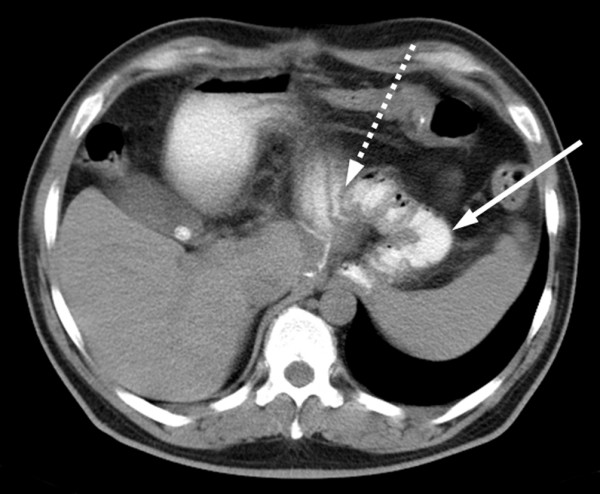
**Postoperative CT-Scan**. Postoperative follow-up CT scan after 18 months showing the jejunal interposition (gastro-jejunostomy: interrupted arrow).

The postoperative course was uneventful, the patient recovered quickly. He was allowed regular food intake from day four onward could be discharged from hospital at the 10th postoperative day. After recovery the patient continued antiproliverative therapy with imatinib mesylate at 400 mg per day. One and a half years later he is in an excellent physical condition and free from disease. The patient reports no restriction in the oral food uptake nor regurgitation or sourness. CT imaging and abdominal ultrasound did not show recurrent or metastatic tumor growth (figure 6).

## Discussion

First reported in 1998, most GISTs are characterized by an oncogenic mutation in the Kit tyrosine kinase (CD 117), allowing spontaneous (ligand-independent) receptor dimerization and kinase activation [13,14]. The c-Kit expression distinguishes GISTs from tumors of smooth muscle cells. GISTs are the most common non-epithelial tumors of the GI tract. The diagnosis of GIST has dramatically increased since 1992, and survival has dramatically improved since 2002. Size and mitotic count are the most prognostic features and are used for risk stratification [15]. All tumors larger than 2 cm have a risk of recurrence and tumor exceeding 5 cm should be considered potentially malignant.

Initial treatment of localized GIST should aim at complete resection of the tumor with margins of 1–2 cm. Segmental resection of small bowel or colon is adequate, no lymphatic dissection is required. Small tumors (< 3 cm) of the stomach could be excised by a laparoscopic approach [16]. Larger tumors request functional gastric resection like antrectomy or resection of the gastric fundus with tube forming [17]. Locoregionally advanced tumors or those poorly positioned requiring total gastrectomy or other extended resection should be considered for neoadjuvant treatment with imatinib mesylate and afterwards re-evaluated for resection [18,19]. The response rate to be expected from drug therapy is 75% to 80%. It is reasonable to consider the disease as initially as "unresectable" without incurring risk of unacceptable morbidity or functional deficit and therefore to use imatinib mesylate therapy as the first-line anticancer therapy.

In our case, primary resection would have necessitated a multivisceral resection including the distal part of the esophagus, the proximal stomach, spleen and a part of the diaphragm to remove the tumor without contamination and clear margins. After pre-treatment with imatinib mesylate and relevant tumor shrinkage segmental resection was possible. Reconstruction of the upper part of the GI tract after resection of carcinoma of the GEJ usually necessitates esophago-gastrostomy or a Roux-en-Y procedure. Both are often followed by considerable postoperative morbidity mainly due to acidic or biliary esophageal reflux. Dumping syndrome and weight loss are sequelae of excluding the duodenal passage [20]. For locally advanced carcinomas of the esophagus and the GEJ requiring extended lymph node dissection this procedural specific morbidity and mortality is accepted.

Unlike, the resection of GIST tumors does not require lymph node dissection. For these reasons GIST of the GEJ should be treated by limited resection and optimal functional reconstruction if the size of the tumor allow. In 1955, Merendino and Dillard published a technique to reconstruct the esophagogastric passage and to prevent reflux and esophagitis following resection of the GEJ [21]. Initially, the procedure was developed for a variety of benign diseases like severe esophagitis, stricture or cardiospasm. In its final version, the operation consists of the interposition of an isoperistaltic segment of jejunum between the esophagus and stomach, with bilateral vagotomy and a pyloroplasty. In a series of patients who underwent this operation the mean Gastrointestinal Quality of Life Index did not differ from that of healthy controls [22]. We regard the Merendino procedure as an ideal indication for resectable GIST of the proximal stomach or GEJ. In our case, the clinical response to imatinib mesylate with tumor shrinkage prevented an abdomino-thoracic approach with multivisceral resection and allowed a reconstruction of a functional competent GEJ.

Neoadjuvant treatment with imatinib mesylate for locally advanced GIST represents a not yet fully established strategy to handle large GIST that can be resected only with curative intent by major procedures accompanied with organ function loss, i.e. Whipple' procedure for GIST of the duodenum or abdomino-perineal excision for GIST of the rectum or recto-vaginal septum. Two phase II trials currently explore this option in a standardized fashion. The study of the RTOG S-0132 initially used 8 weeks (now 3 months) treatment with imatinib mesylate at 400 mg daily upfront to resection. The so-called Apollon study (CSTI571 BDE43) foresees 6 months of pre-treatment. It is still matter of debate whether treatment with imatinib mesylate should be continued postoperatively. The experience of several centers [[23], BFR14 study] as well as the guidelines of the NCCN recommend a minimum treatment period with imatinib mesylate of 12 months, thus we continued therapy after recovery in our patient. Response to treatment fulfilling the criteria of a partial remission according to RECIST criteria [24] requires 3–6 months of therapy [25]. Therefore special criteria of contrast media uptake in CT or MRI have been established [26,27]. Positron emission tomography with 18F-fluoro-desoxyglucose (FDG) is an ideal tool to monitor treatment effects as it demonstrates shut-down of the tumor metabolism as early as 24 h or 72 hours [28]. In case CT does not show tumor shrinkage early, treatment can be continued safely if PET documents stop of proliferative activity. Also in our patient, ^18^F-FDG PET was antecedent to CT in demonstrating response to imatinib mesylate and allowed us to complete the full course of preoperative therapy. PET otherwise has been used successfully in epithelial cancer of the esophagus to evaluate treatment with preoperative radiochemotherapy [29].

## Conclusion

Combined modality therapy of preoperative imatinib mesylate downstaging of a GIST of the GEJ with limited resection and reconstruction by a interposition of a jejunal segment resulted in an R0 resection and excellent functional outcome of the patient.

## Competing interests

Peter Hohenberger has received research grants from Novartis. All other authors do not have a financial or personal relationship with a commercial entity that has an interest in the subject of this manuscript.

## Authors' contributions

WS wrote the manuscript and carried out literature review. UR contributed to data management and preparing of the manuscript. GK did the endoscopy work-up and biopsies. HUS did the molecular pathology work-up. ADS did the PET imaging. MS conceived the idea, did supervision of manuscript preparation and proof reading. PH initiated treatment, did surgical procedures and approved the final version of the paper. All authors read and approved the final manuscript.
